# Supplementary material for: Mitochondrial injury induced by a Salmonella genotoxin triggers the proinflammatory senescence-associated secretory phenotype
Source: Nat Commun. 2024 Mar 30;15:2778. doi: 10.1038/s41467-024-47190-y (PMC10981749; doi:10.1038/s41467-024-47190-y)

Supplementary information

Mitochondrial injury induced by a *Salmonella* genotoxin triggers the proinflammatory senescence-associated secretory phenotype

Han-Yi Chen[1], Wan-Chen Hsieh[2], Yu-Chieh Liu[1], Huei-Ying Li[3], Po-Yo Liu[1], Yu-Ting Hsu[1], Shao-Chun Hsu[4], An-Chi Luo[4], Wei-Chen Kuo[5], Yi-Jhen Huang[6], Gan-Guang Liou[7], Meng-Yun Lin[8], Chun-Jung Ko[8], Hsing-Chen Tsai[6,9,10], and Shu-Jung Chang[1]*

[1]Graduate Institute of Microbiology, College of Medicine, National Taiwan University, Taipei, Taiwan.

[2]Institute of Molecular and Cellular Biology, National Tsing Hua University, Hsinchu, Taiwan.

[3]Medical Microbiota Center of the First Core Laboratory, College of Medicine, National Taiwan University, Taipei, Taiwan.

[4]Imaging Core, College of Medicine, National Taiwan University, Taipei, Taiwan.

[5]Institute of Biochemistry and Molecular Biology, College of Medicine, National Taiwan University, Taipei, Taiwan.

[6]Graduate Institute of Toxicology, College of Medicine, National Taiwan University, Taipei, Taiwan.

[7]Cryo-EM Core, College of Medicine, National Taiwan University, Taipei, Taiwan.

[8]Graduate Institute of Immunology, College of Medicine, National Taiwan University, Taipei, Taiwan.

[9]Department of Internal Medicine, National Taiwan University Hospital, Taipei, Taiwan.

[10]Center for Frontier Medicine, National Taiwan University Hospital, Taipei, Taiwan.

For correspondence: sjchang@ntu.edu.tw

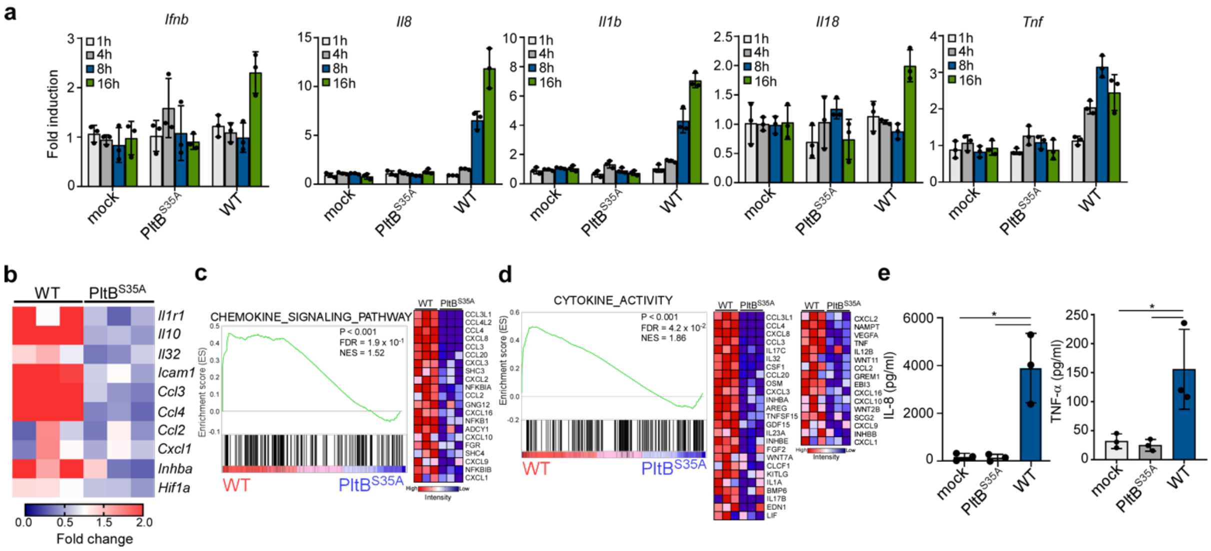

Supplementary Figure 1. **Typhoid toxin induces cellular senescence and proinflammatory SASP in THP-1-derived macrophages. a**, RT-qPCR analysis of mRNA levels of indicated genes in WT typhoid toxin and PltB^{S35A} mutant toxin-treated THP-1-derived macrophages (n = 3 independent experiments). **b**, Heatmap analysis depicting mRNA levels of cellular senescence-related genes determined by RT-qPCR in WT typhoid toxin and PltB^{S35A} mutant toxin-treated macrophages (n = 3 independent experiments). **c,d**, Gene set enrichment analysis showing upregulated genes associated with chemokine signaling pathway (**c**) and cytokine activity (**d**) in WT typhoid toxin-treated macrophages compared to PltB^{S35A} mutant-treated macrophages. **e**, Assessment of IL-8 and TNF-α levels in THP-1-derived macrophages exposed to mock, WT typhoid toxin and the PltB^{S35A} mutant using ELISA. Data are presented as mean ± s.d of three independent experiments. Statistical analysis was performed using unpaired two-sided *t*-tests; *$P < 0.05$. Source data are provided as a Source Data file.

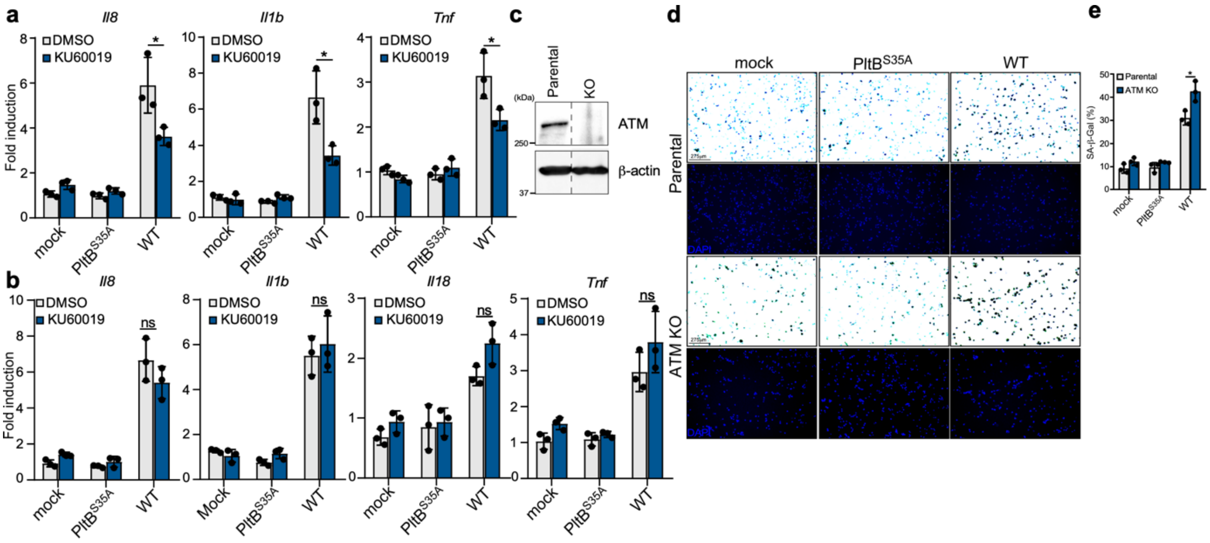

Supplementary Figure 2. **The role of ATM in typhoid toxin-induced cellular senescence and proinflammatory SASP in THP-1-derived macrophages. a,b**, THP-1-derived macrophages were exposed to typhoid toxin at 37 °C for 1 hour and then changed to regular growth medium with or without 1 μM of the ATM inhibitor (KU60019). Total RNA was purified at 8 (**a**) and 16 (**b**) hours post-treatment, and the mRNA levels of indicated genes were analyzed using RT-qPCR (n=3 independent experiments). **c**, Western blot analysis of ATM-deficient THP-1-derived macrophages. β-actin as a loading control. Dotted lines marks places where experimentally relevant lanes were joined. **d,e**, SA-β-gal activity staining in the parental and ATM-deficient THP-1-derived macrophages exposed to typhoid toxin (**d**). The quantification results (**e**) are presented as mean ± s.d (n=3). Statistical analysis was performed using unpaired two-sided *t*-tests; *$P < 0.05$. Source data are provided as a Source Data file.

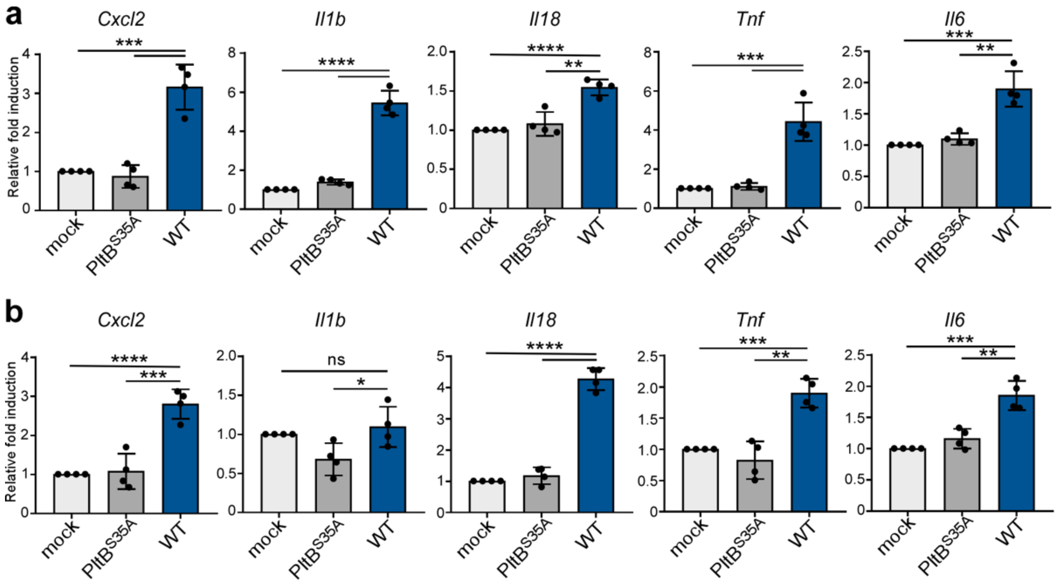

Supplementary Figure 3. **Expression of proinflammatory components in different subset of murine bone marrow-derived macrophages.** RT-qPCR analysis of mRNA levels in primary M1(**a**) and M2 (**b**) macrophages exposed to mock, WT typhoid toxin, and the PltB^{S35A} mutant. Data are presented as mean ± s.d (n=4). Statistical analysis was performed using unpaired two-sided *t*-tests; *$P < 0.05$, **$P < 0.01$, ***$P < 0.001$, ****$P < 0.0001$ ns, not significant ($P > 0.05$). Source data are provided as a Source Data file.

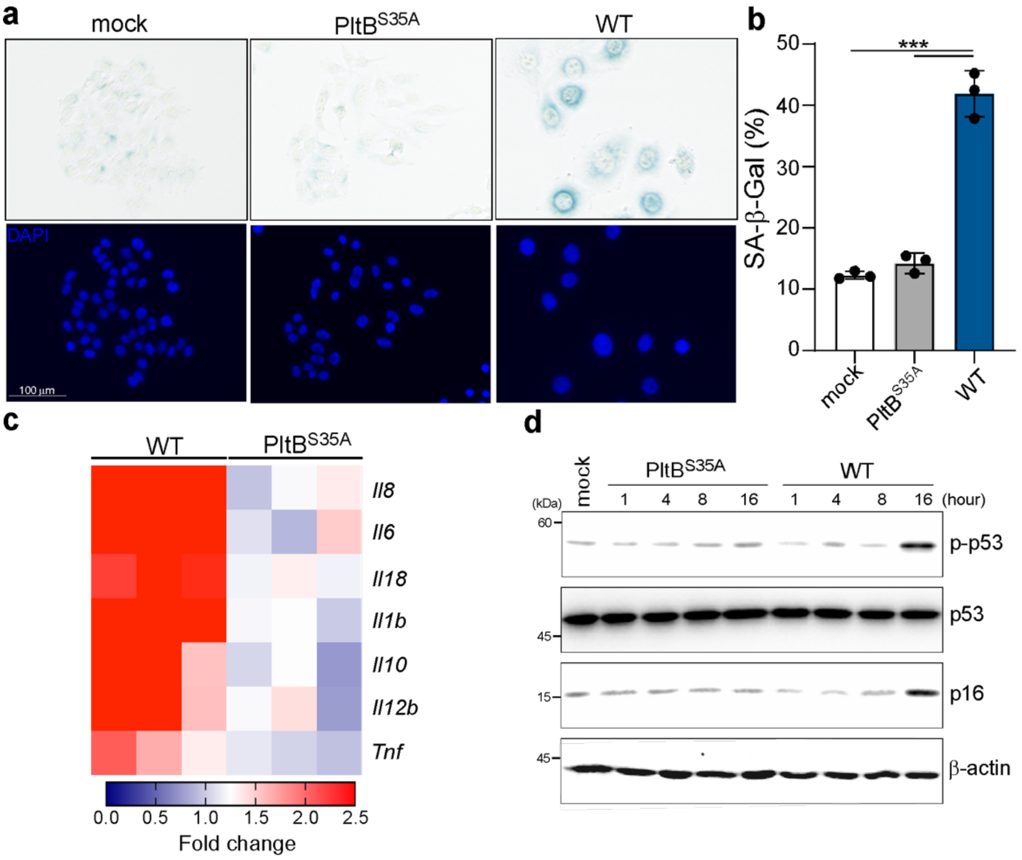

Supplementary Figure 4. Typhoid toxin induces cellular senescence and the proinflammatory SASP in Henle-407 cells. a,b, SA-β-gal activity staining in Henle-407 cells at 48 hours post-typhoid toxin treatment (**a**). The quantification results (**b**) are presented as mean ± s.d (n=3). Statistical analysis was performed using unpaired two-sided *t*-tests; ***$P < 0.001$. **c**, Heatmap analysis depicting mRNA levels of cellular senescence-related genes determined by RT-qPCR in WT typhoid toxin and PltB^{S35A} mutant toxin-treated Henle-407 cells (n = 3 independent experiments). **d**, Henle-407 cells were exposed to typhoid toxin at 37 °C for 1 hour and then changed to regular growth medium. The cell lysates were collected at indicated time points and subjected to western blot analysis for protein assessment. Source data are provided as a Source Data file.

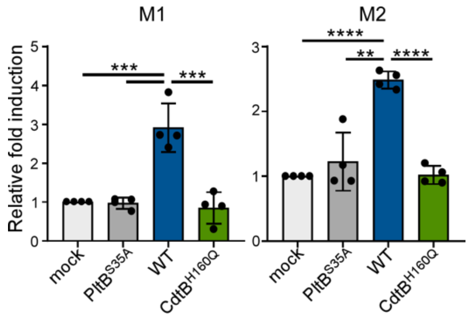

Supplementary Figure 5. **The mRNA level of *Ifnb* in different subset of murine bone marrow-derived macrophages.** Primary M1 and M2 macrophages were treated with WT typhoid toxin and the PltB^{S35A} mutant, and the mRNA level of *Ifnb* was analyzed using RT-qPCR. Data are presented as mean ± s.d (n=4). Statistical analysis was performed using unpaired two-sided *t*-tests; ***P* < 0.01, ****P* < 0.001, *****P* < 0.0001. Source data are provided as a Source Data file.

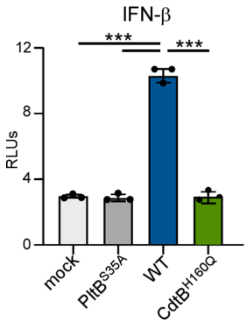

Supplementary Figure 6. **IFN-β luciferase reporter activity in Henle-407 epithelial cells.** IFN-β luciferase reporter activity in cells exposed to WT typhoid toxin, the PltB^{S35A} and the CdtB^{H160Q} mutants at 37 °C for 1 hour and then changed to regular growth medium. The reporter activity was measured at 16 hours post-treatment. Data are represented as the mean ± s.d (n=3). Statistical analysis was performed using unpaired two-sided *t*-tests; ***$P < 0.001$. Source data are provided as a Source Data file.

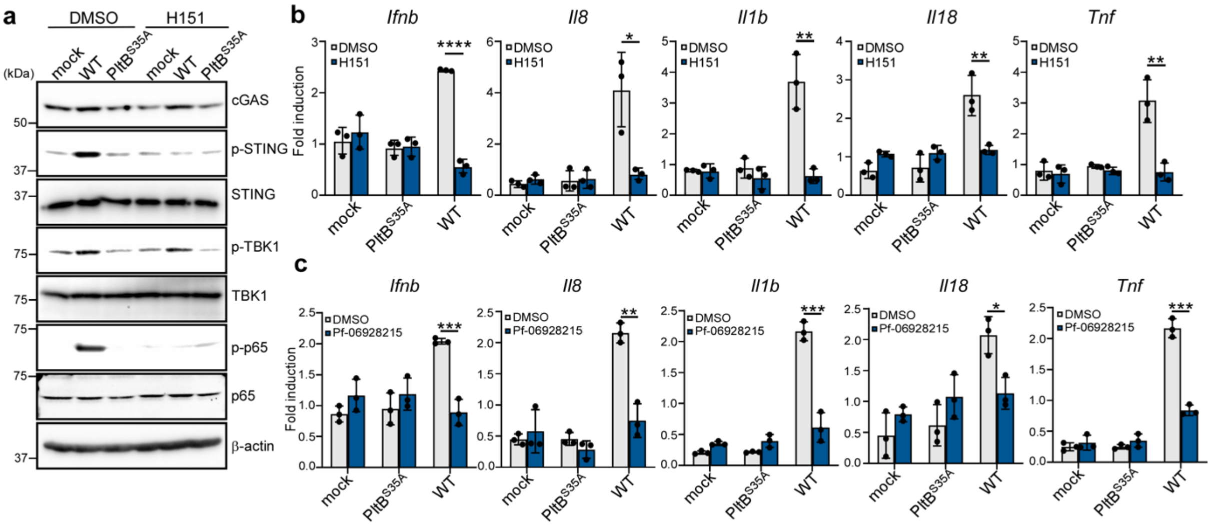

Supplementary Figure 7. **Inhibition of cGAS-STING signaling reduces the expression fo proinflammatory components in cells. a**, Western blot analysis of THP-1-derived macrophages exposed to typhoid toxin with presence of the STING inhibitor, H151 (0.5 μM). **b,c**, RT-qPCR analysis of indicated genes in typhoid toxin-treated THP-1-derived macrophages with the presence of the STING inhibitor (**b**) or 25 μM of the cGAS inhibitor (**c**). Data are represented as the mean ± s.d (n=3). Statistical analysis was performed using unpaired two-sided *t*-tests; **P* < 0.05, ***P* < 0.01, ****P* < 0.001, *****P* < 0.0001. Source data are provided as a Source Data file.

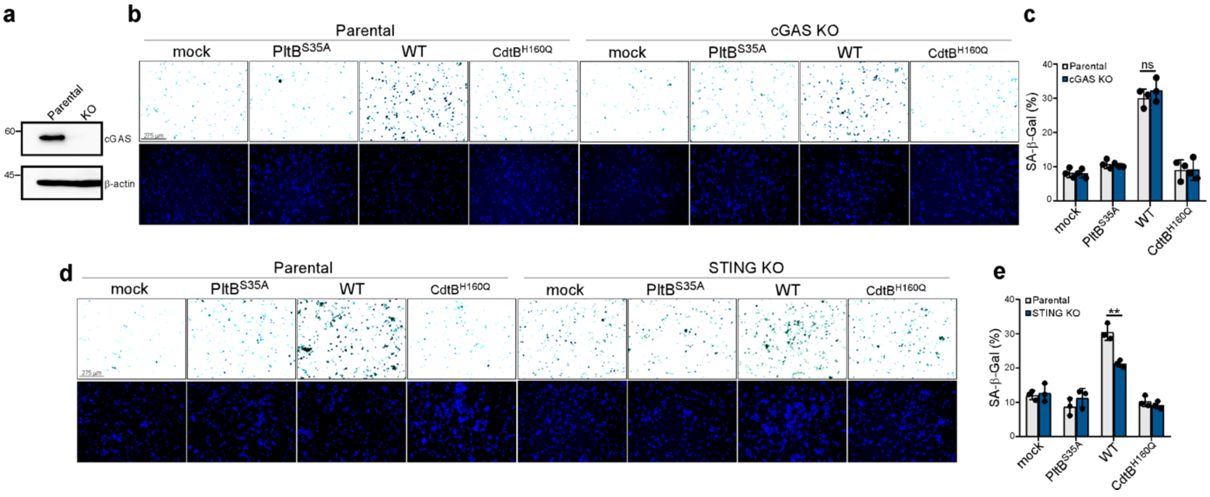

Supplementary Figure 8. The role of the cGAS-STING axis in cellular senescence induced by typhoid toxin. a, Western blot analysis was performed on cGAS-deficient THP-1-derived macrophages and the parental cells to examine cGAS protein expression. **b-e**, The parental THP-1-derived macrophages and knockout (KO) cells were exposed to wild-type (WT) typhoid toxin or its mutants at 37 °C for 1 hour and then changed to regular growth medium. SA-β-gal activity staining was performed at 48 hours post-treatment in the parental THP-1-derived macrophages and those deficient in cGAS (**b, c**), as well as in cells lacking STING (**d, e**). The quantification results are presented as mean ± s.d with a minimum of 100 cells counted (n=3). Statistical analysis was performed using unpaired two-sided *t*-tests; **, $P < 0.01$, ns indicates no significant difference ($P > 0.05$). Source data are provided as a Source Data file.

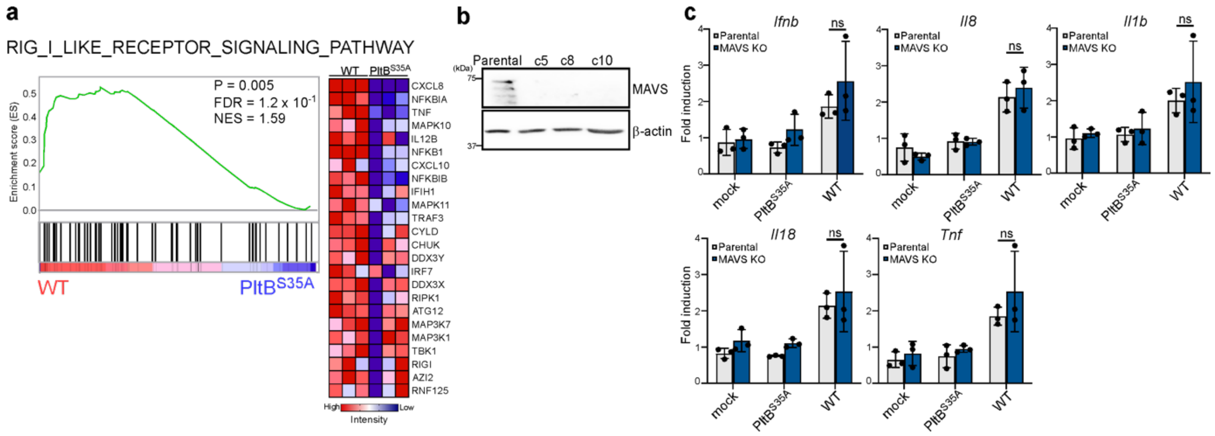

Supplementary Figure 9. **RIG-I-MAVS signaling is not involved in the SASP induced by typhoid toxin. a**, Gene set enrichment analysis of upregulated genes associated with the RIG-I-like receptor signaling pathway in wild-type (WT) typhoid toxin-treated macrophages compared to PltB^{S35A} mutant-treated macrophages. **b,c**, Western blot analysis was performed on MAVS-deficient THP-1-derived macrophages and their parental cells to examine MAVS protein expression (**b**). The MAVS-deficient knockout (KO) clone 8 (c8) exposed to typhoid toxin was further analyzed by RT-qPCR to evaluate the mRNA levels of proinflammatory genes (**c**). Data are presented as mean ± s.d (n=3). Statistical analysis was conducted using unpaired two-sided *t*-tests; ns indicates no significant difference ($P > 0.05$). Source data are provided as a Source Data file.

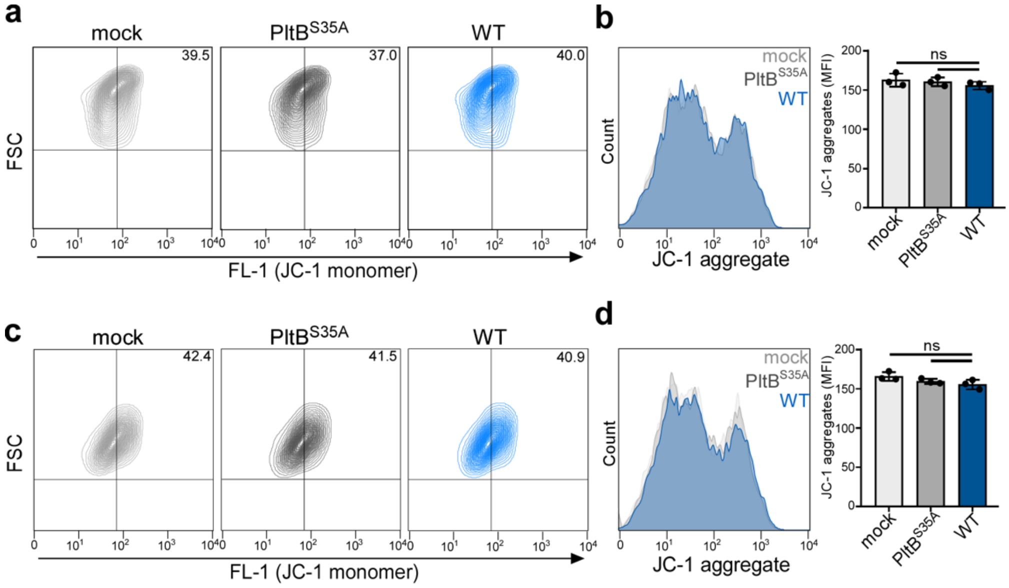

Supplementary Figure 10. **Mitochondrial membrane potential measured by flow cytometry.** THP-1-derived macrophages were exposed to typhoid toxin at 37 °C for 1 hour and then changed to regular growth medium. At 3 (**a,b**) and 8 (**c,d**) hours after typhoid toxin treatment, the cells were stained with JC-1 dye and analyzed using flow cytometry. Histogram plots (**b, d**) display JC-1 intensity, and the quantification of JC-1 aggregate fluorescence served as an indicator of normal mitochondrial membrane potential. Data are presented as the mean ± s.d (n=3). Statistical analysis was performed using unpaired two-sided *t*-tests; ns, not significant. Source data are provided as a Source Data file.

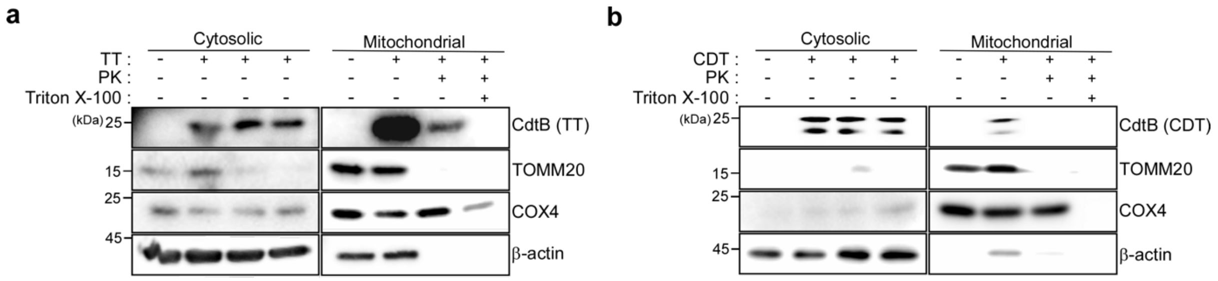

Supplementary Figure 11. **Localization of typhoid toxin in the mitochondria.** Western blot analysis of proteinase K digestion of mitochondrial fractions in THP-1-derived macrophages exposed to typhoid toxin (TT) (**a**) or cytolethal distending toxin (CDT) (**b**). Mitochondrial fractions obtained from cells were digested with 12.5 μg/ml proteinase K (PK) in the buffer containing 1% Triton X-100 for 30 minutes at 37°C. Proteins were detected by western blot analysis using antibodies against CdtB (typhoid toxin), CdtB-6xHis (CDT), TOMM20 (an outer mitochondrial membrane protein), COX4 (an inner mitochondrial membrane protein) and β-actin as a loading control.

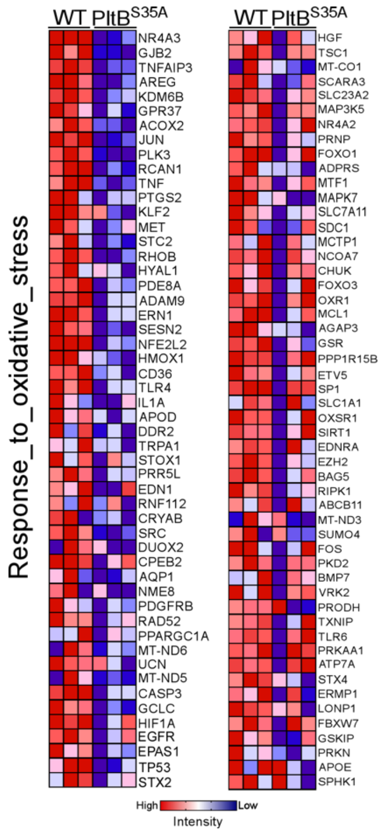

Supplementary Figure 12. **Induction of oxidative stress response by typhoid toxin in macrophages.** Heatmap showing upregulated genes associated with oxidative stress in wild-type (WT) typhoid toxin-treated macrophages compared with PltB^{S35A} mutant-treated macrophages.

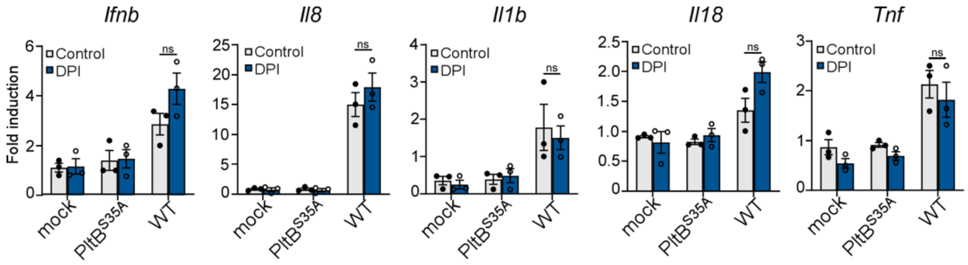

Supplementary Figure 13. **NADPH oxidases-generated ROS is not involved in typhoid toxin- induced proinflammatory component expression.** RT-qPCR of indicated genes in THP-1-derived macrophages exposed to WT typhoid toxin and its toxin mutants with or without DPI treatment (2 mM). Data are presented as the mean ± SEM (n=3). Statistical analysis was performed using unpaired two-sided *t*-tests; ns, not significant ($P > 0.05$). Source data are provided as a Source Data file.

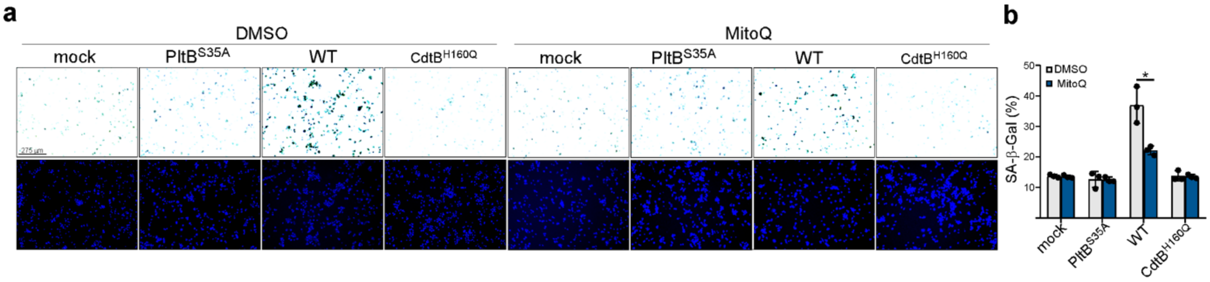

Supplementary Figure 14. Suppression of mitochondrial ROS production mitigates SA-β-Gal activity. SA-β-gal activity staining was performed 48 hours after typhoid toxin treatment in the THP-1-derived macrophages with or without MitoQ (2 μM) (**a**). The quantification results are presented as mean ± s.d (n=3) with a minimum of 100 cells counted (**b**). Statistical analysis was performed using unpaired two-sided *t*-tests; *, $P < 0.05$. Source data are provided as a Source Data file.

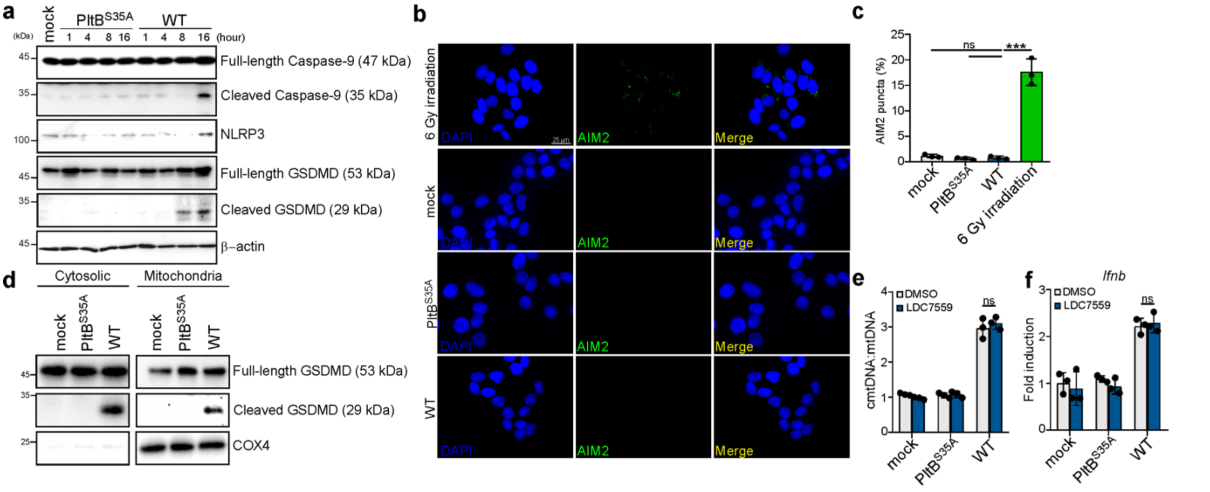

Supplementary Figure 15. **The role of GSDMD in mitochondrial DNA release to the cytosol. a,** Analysis of caspase-9, NLRP3, and GSDMD activation in THP-1-derived macrophages. Cell lysate was collected at 16 hours post-treatment with wild-type (WT) typhoid toxin or the PltB^{S35A} mutant, and subjected to western blot analysis. **b**,**c**, Henle-407 cells were treated either WT typhoid toxin or the PltB^{S35A} mutant, and then fixed for immunostaining at 16 hours post-treatment (**b**). As a positive control to visualize AIM2 inflammasome specks, cells were exposed to irradiation. Quantification results are presented in (**c**) with a minimum of 300 cells counted. **d**, Assessment of N-GSDMD mitochondrial association in THP-1-derived macrophages treated with typhoid toxin using mitochondrial fractionation. **e**, RT-qPCR of cytosolic mtDNA (cmtDNA) quantified relative to total mitochondrial DNA (mtDNA) in THP-1-derived macrophages exposed to typhoid toxin with treatment of the GSDMD inhibitor (LDC7559) (1μM). Data are presented as the mean ± s.d (n=3). **f**, RT-qPCR of *Ifnb* mRNA levels in THP-1-derived macrophages exposed to WT typhoid toxin and its toxin mutants with or without the GSDMD inhibitor. Data are presented as the mean ± s.d (n=3). Statistical analysis was performed using unpaired two-sided *t*-tests; ***, $P < 0.001$, ns, not significant ($P > 0.05$). Source data are provided as a Source Data file.

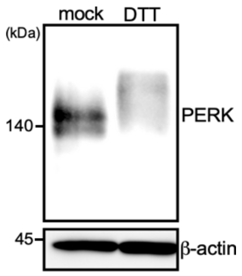

Supplementary Figure 16. **Assessment of PERK activation by western blot analysis.** Cell lysates were subjected to western blot analysis using antibodies against PERK and β-actin as a loading control. To detect the band shift in the mobility of PERK (PERK activation), cells were treated with DTT for 30 minutes prior to harvesting for western blot analysis.

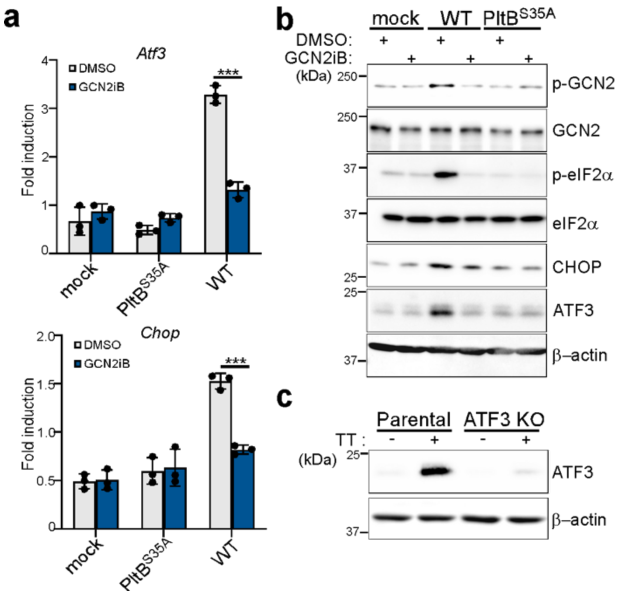

Supplementary Figure 17. **GCN2 inhibition suppressed the integrated stress response induced by typhoid toxin.** THP-1-derived macrophages exposed to typhoid toxin were treated with the GCN2 inhibitor (GCN2iB) (0.5 μM) and subjected to RT-qPCR analysis (**a**) and western blot analysis (**b**). The mRNA level data are presented as mean ± s.d (n=3). Statistical analysis was conducted using unpaired two-sided *t*-tests; *** $P < 0.001$. **c**, Western blot analysis of ATF3-deficient THP-1-derived macrophages. ATF3-deficient THP-1-derived macrophages were generated using CRISPR/Cas9 genome-editing technology, and the successful knockout cell line exposed to wild-type typhoid toxin was confirmed by western blot analysis using an antibody against ATF3, with β-actin serving as a loading control. Source data are provided as a Source Data file.

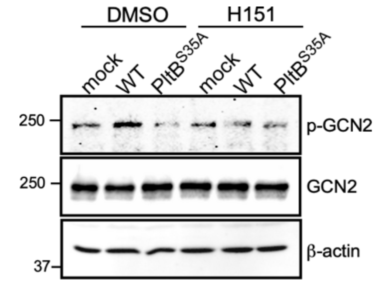

Supplementary Figure 18. **Western blot analysis of macrophages treated with typhoid toxin in the presence of the STING inhibitor.** THP-1-derived macrophages were exposed to typhoid toxin and treated with the STING inhibitor, H151 (0.5 μM). Cell lysates were subjected to western blot analysis using an antibody against phosphorylated GCN2, and β-actin was used as a loading control.

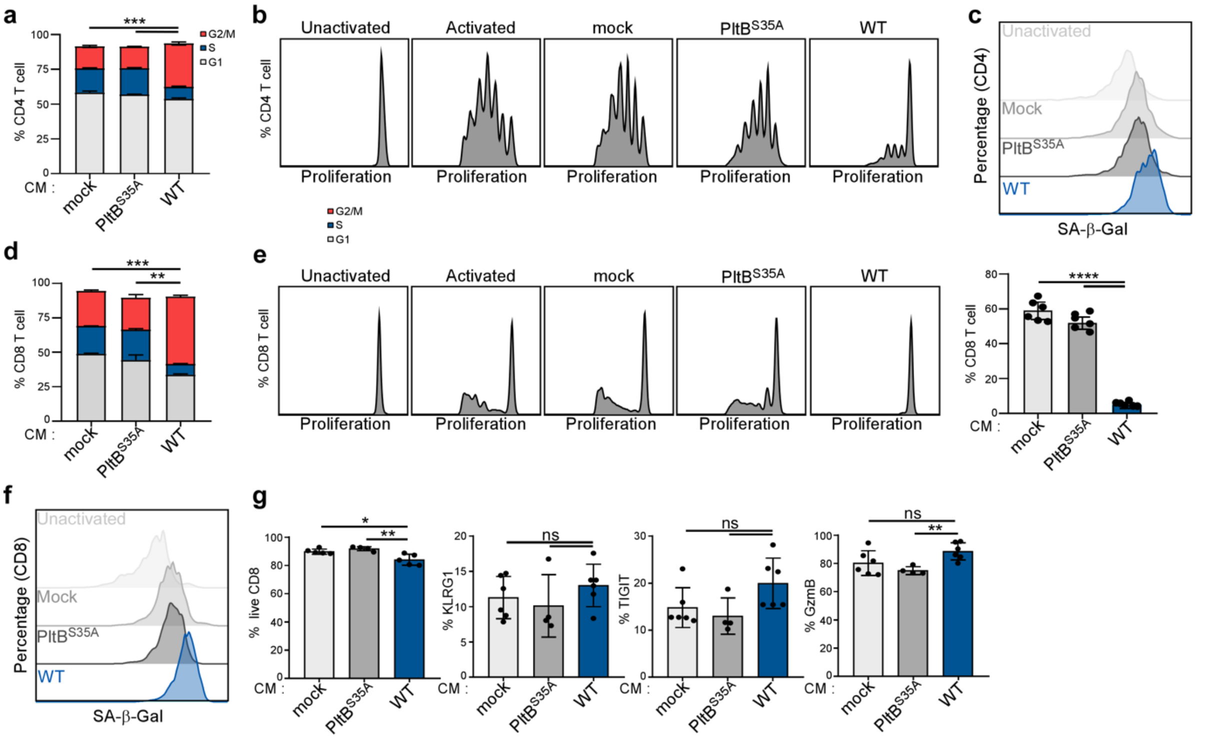

Supplementary Figure 19. **Paracrine effect of cellular senescence on T cells**. **a,d**, Cell cycle analysis of CD4 T (**a**) and CD8 T (**d**) cells incubated with conditioned medium (CM) from typhoid toxin-treated RAW 264.7 macrophages. Data are presented as the mean ± s.d (n=3). **b**,**e** CellTrace Violet profiles for CD4 (**b**) and CD8 T (**e**) cells are presented with percentages indicating the proportion of cells over 5 days. **c**,**f**, Measurement of SA-β-Gal activity in CD4 T (**c**) and CD8 T (**f**) cells. **g**, Flow cytometry analysis of cell viability, and the expression of cell surface markers including KLRG1 ($CD8^+KLRG1^+$), TIGIT ($CD8^+TIGIT^+$), and granzyme B ($CD8^+GzmB^+$) in CD8 T cells. Data are presented as the mean ± s.d (n=3). Statistical analysis was performed using unpaired two-sided *t*-tests; **P* < 0.05, ***P* < 0.01, ****P* < 0.001, *****P* < 0.0001, ns, not significant (*P* > 0.05). Source data are provided as a Source Data file.

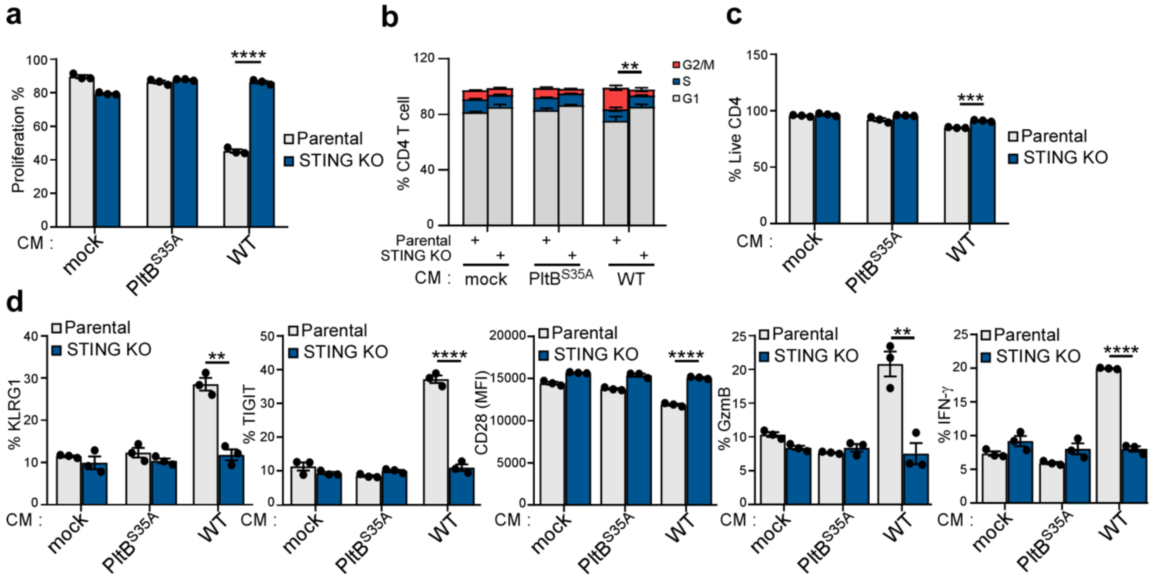

Supplementary Figure 20. **The role of STING in paracrine cellular senescence effect on CD4 T cells.** Proliferation assay (**a**), cell cycle analysis (**b**), cell viability (**c**), and expression of cell surface markers, including KLRG1, TIGIT, CD28, granzyme B, and IFN-γ (**d**) of CD4 T cells incubated with conditioned medium (CM) from typhoid toxin-treated parental or STING knockout (KO) RAW264.7 macrophages. Data are presented as the mean ± s.d (n=3). Statistical analysis was performed using unpaired two-sided *t*-tests; $**P < 0.01$, $***P < 0.001$, $****P < 0.0001$. Source data are provided as a Source Data file.

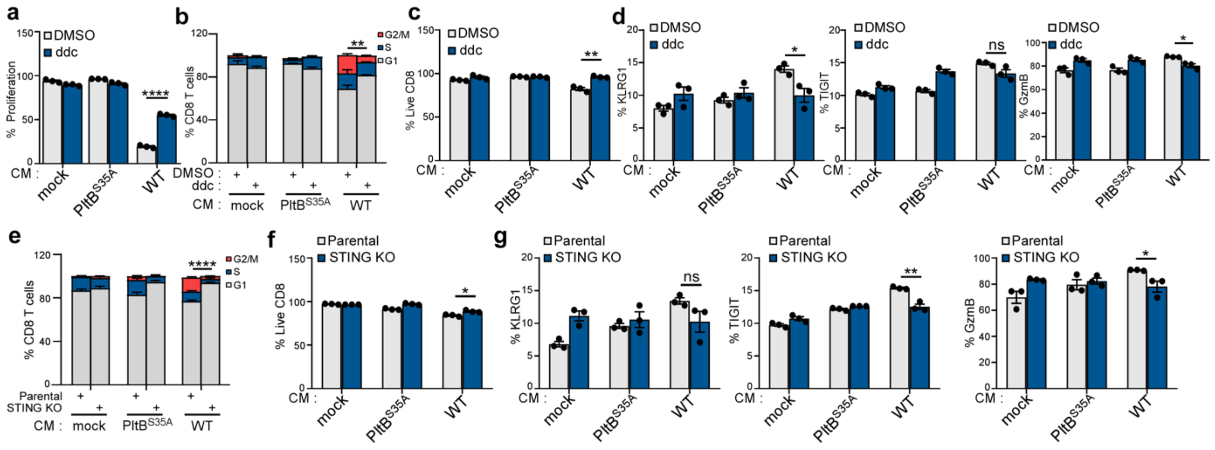

Supplementary Figure 21. The role of mitochondrial DNA and STING in paracrine cellular senescence effect on CD8 T cells. a-d, Proliferation assay (**a**), cell cycle analysis (**b**), cell viability (**c**), and expression of cell surface markers, including KLRG1, TIGIT, CD28, granzyme B, and IFN-γ (**d**) of CD8 T cells incubated with conditioned medium (CM) from typhoid toxin-treated RAW264.7 macrophages with or without ddC treatment. Data are presented as the mean ± s.d (n=3). **e-g**, Cell cycle analysis (**e**), cell viability (**f**), and expression of cell surface markers (**g**) of CD8 T cells incubated with conditioned medium from typhoid toxin-treated parental or STING knockout (KO) RAW264.7 macrophages. Data are presented as the mean ± s.d (n=3). Statistical analysis was performed using unpaired two-sided *t*-tests; *$P < 0.05$, **$P < 0.01$, ****$P < 0.0001$, ns, not significant ($P > 0.05$). Source data are provided as a Source Data file.

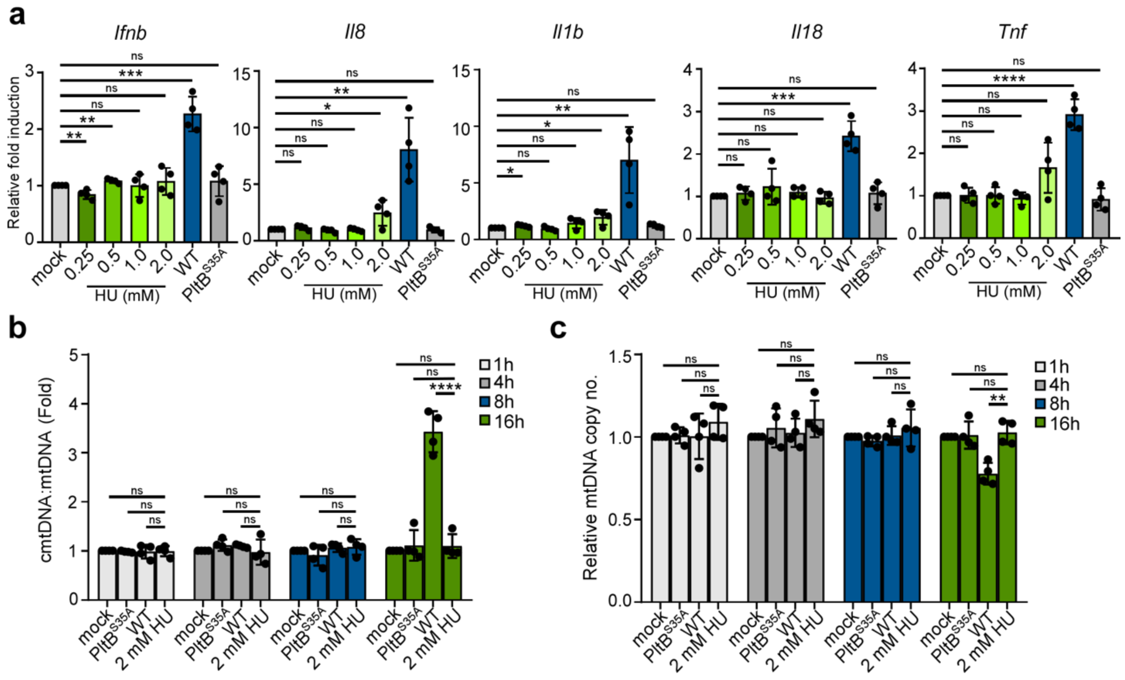

Supplementary Figure 22. Absence of proinflammatory factor expression and mitochondrial damage induction by hydroxyurea. a, THP-1-derived macrophages were treated with hydroxyurea (HU), WT typhoid toxin, and the $PltB^{S35A}$ mutant at 37°C for 1 hour, and then changed to regular growth medium. Total RNA was collected at indicated time points following treatment, and the expression of specific genes was analyzed using RT-qPCR. Data are presented as mean ± s.d (n=4). **b**, RT-qPCR of cytosolic mtDNA (cmtDNA) relative to total mtDNA in THP-1-derived macrophages exposed to hydroxyurea or typhoid toxin using the mt16S primer set. Data are presented as the mean ± s.d from four independent experiments. **c,** RT-qPCR of total mtDNA in THP-1-derived macrophages exposed to hydroxyurea or typhoid toxin. Mitochondrial DNA copy number was normalized by total nuclear DNA (*ACTB*). Data are presented as the mean ± s.d (n=4). Statistical analysis was performed using unpaired two-sided *t*-tests; $*P < 0.05$, $**P < 0.01$, $***P < 0.001$, $****P < 0.0001$ ns, not significant ($P > 0.05$). Source data are provided as a Source Data file.

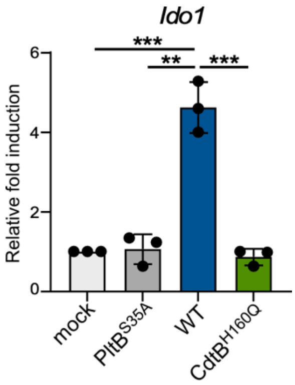

Supplementary Figure 23. **Induction of *Ido1* mRNA expression following the treatment of typhoid toxin**. THP-1-derived macrophages were exposed to wild-type (WT) typhoid toxin, as well as the $PltB^{S35A}$ or $CdtB^{H160Q}$ mutants. Total mRNA was extracted and analyzed by RT-qPCR analysis to determine the expression level of *Ido1* mRNA. Data are presented as mean ± s.d (n=3). Statistical analysis was performed using unpaired two-sided *t*-tests; $^{**}P < 0.01$, $^{***}P < 0.001$. Source data are provided as a Source Data file.

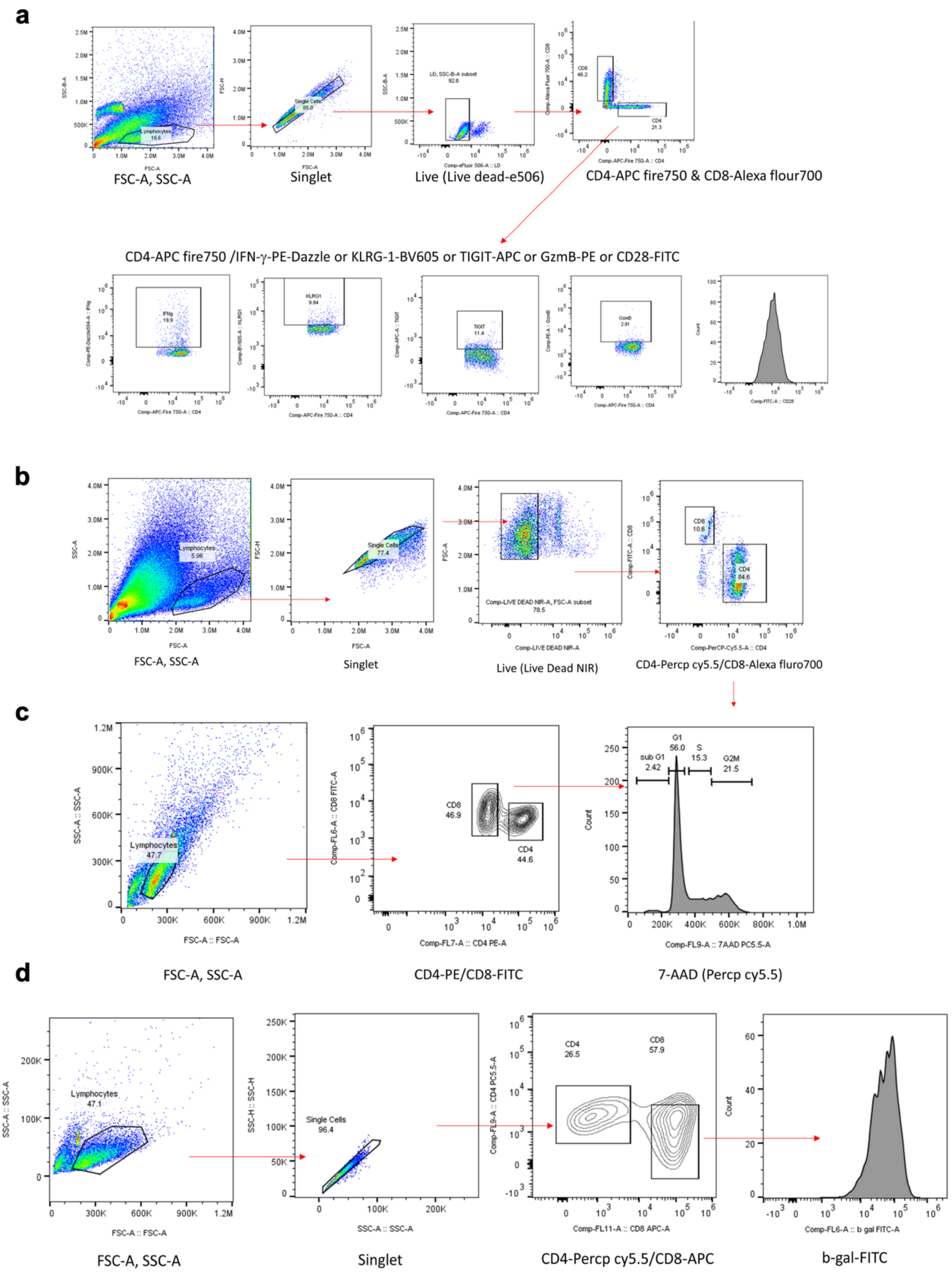

Supplementary Figure 24. Flow cytometry gating strategy for senescence marker on CD4 T cells (a), proliferation on CD4 T cells (b). cell cycle (c), and SA-β-activity (d).

Supplementary figures : Uncropped Western blots

Uncropped blots in Supplementary figure 2 c.

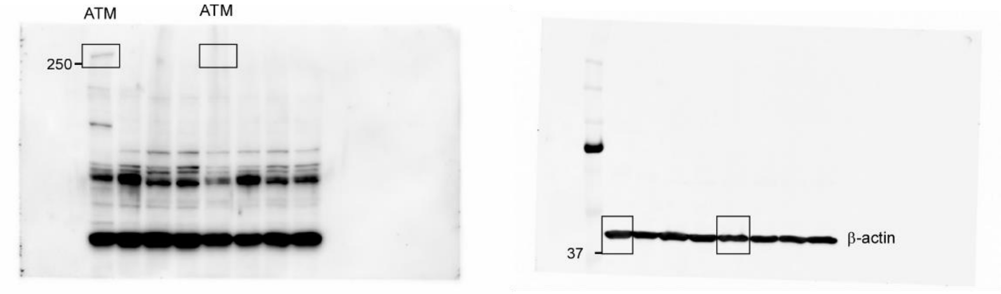

Uncropped blots in Supplementary figure 4 d.

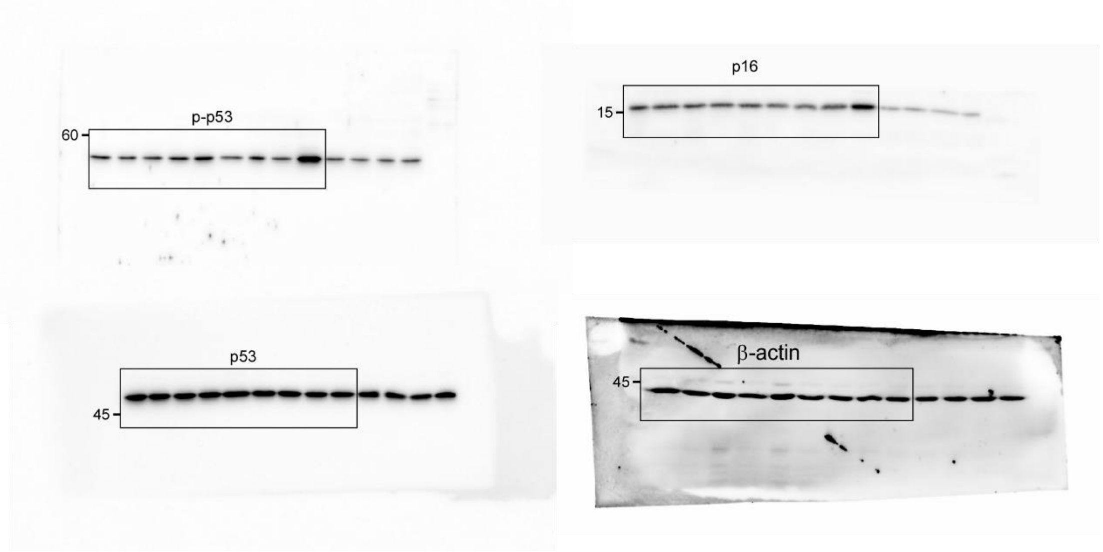

Uncropped blots in Supplementary figure 7 a.

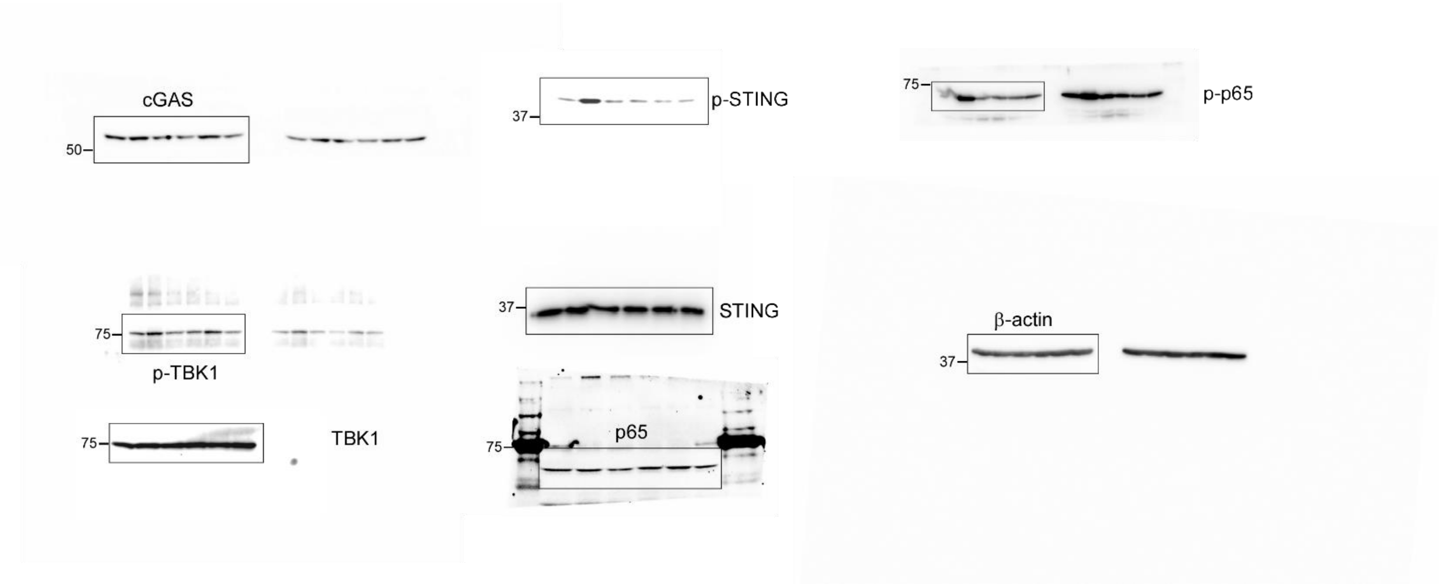

Uncropped blots in Supplementary figure 8 a.

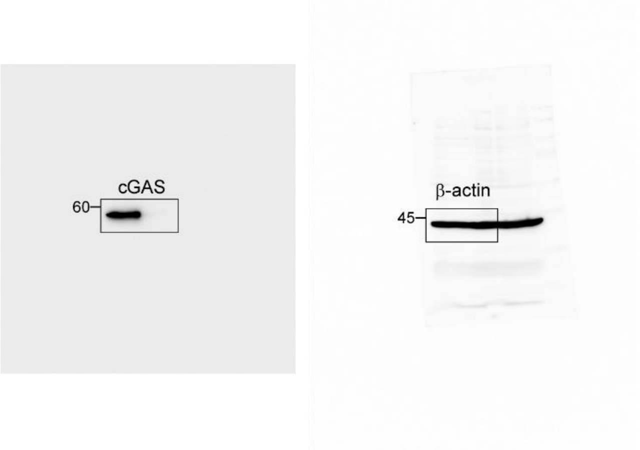

Uncropped blots in Supplementary figure 9.

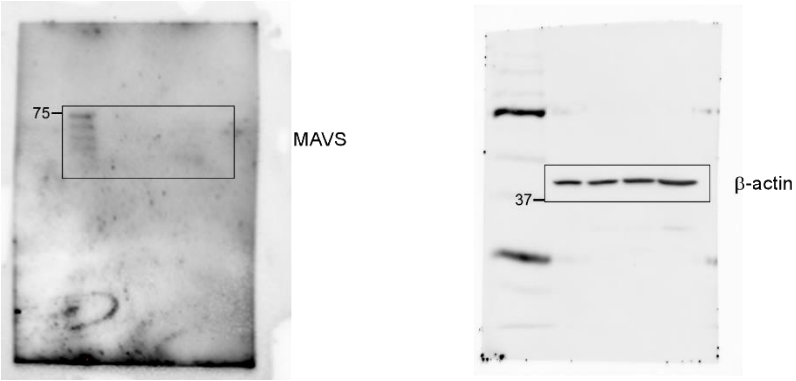

Uncropped blots in Supplementary figure 11 a.

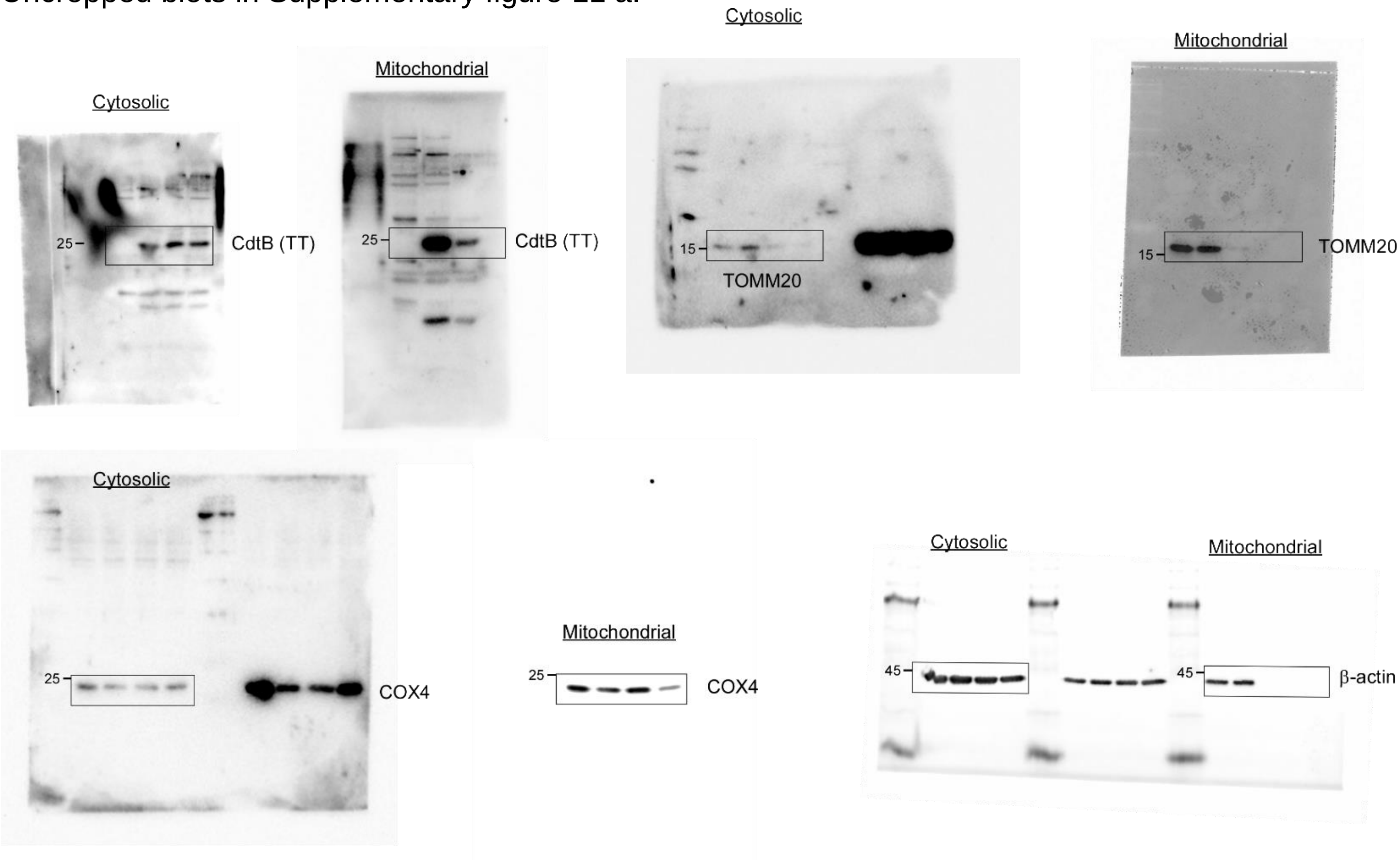

Uncropped blots in Supplementary figure 11 b.

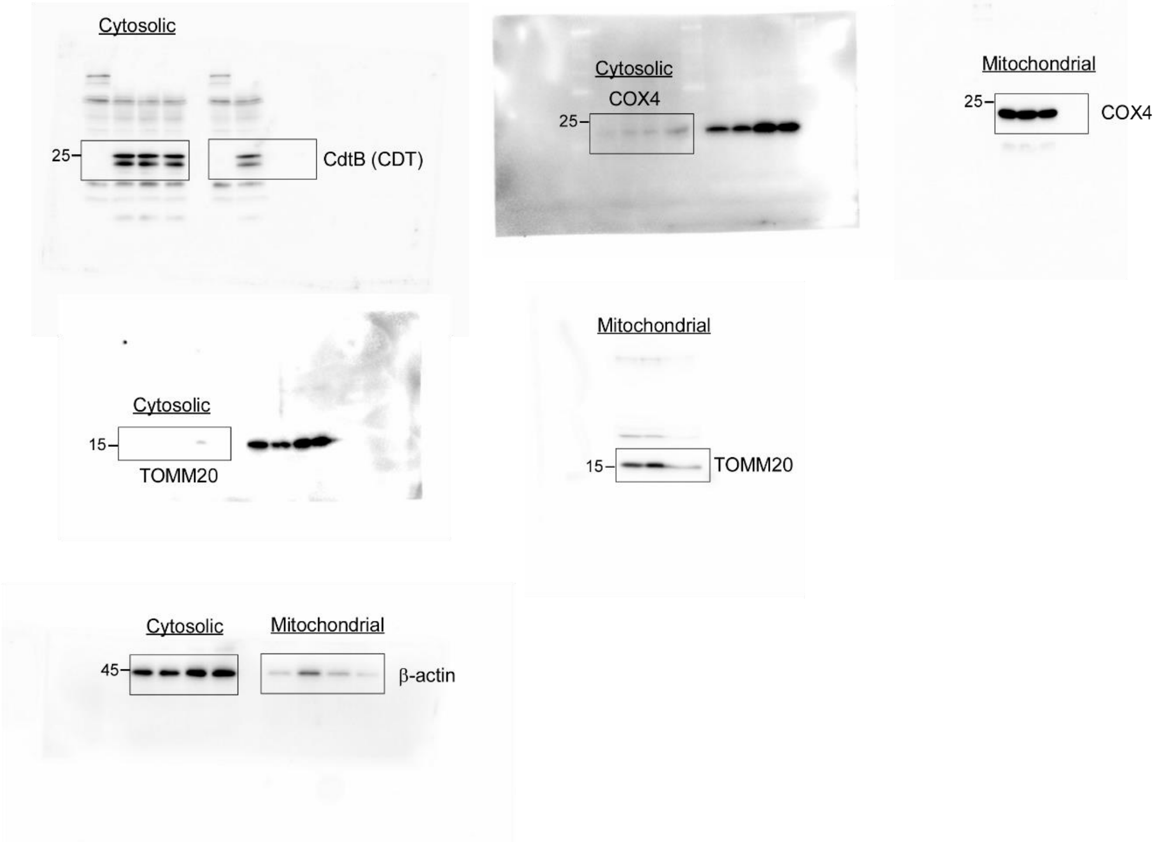

Uncropped blots in Supplementary figure 15 a.

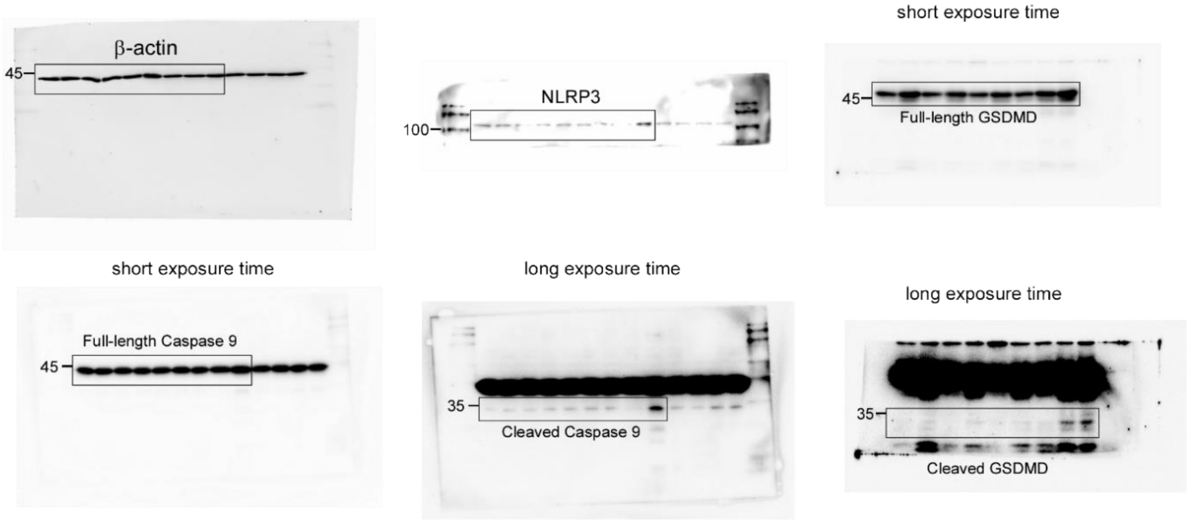

Uncropped blots in Supplementary figure 15 d.

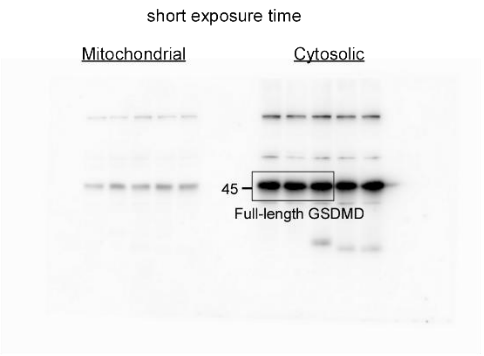

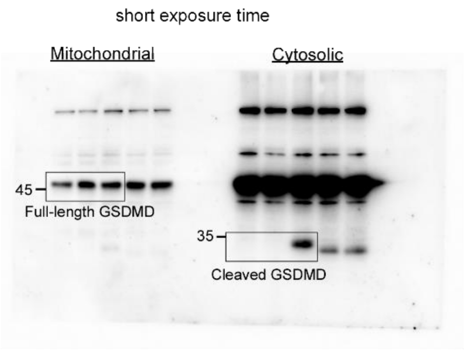

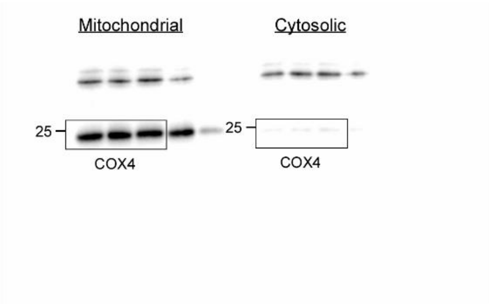

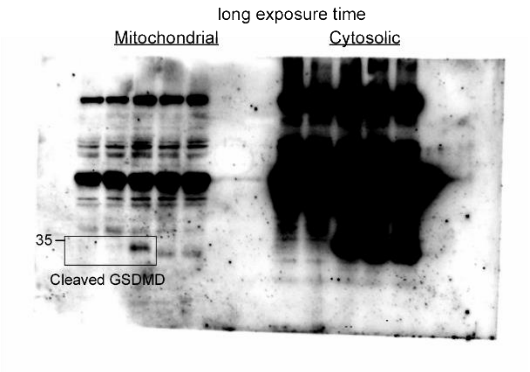

Uncropped blots in Supplementary figure 16.

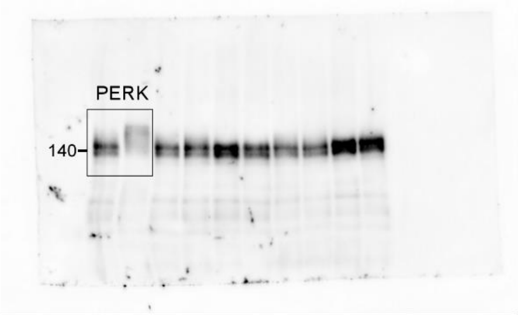

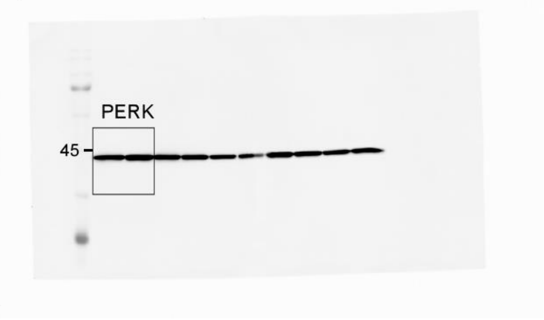

Uncropped blots in Supplementary figure 17 b.

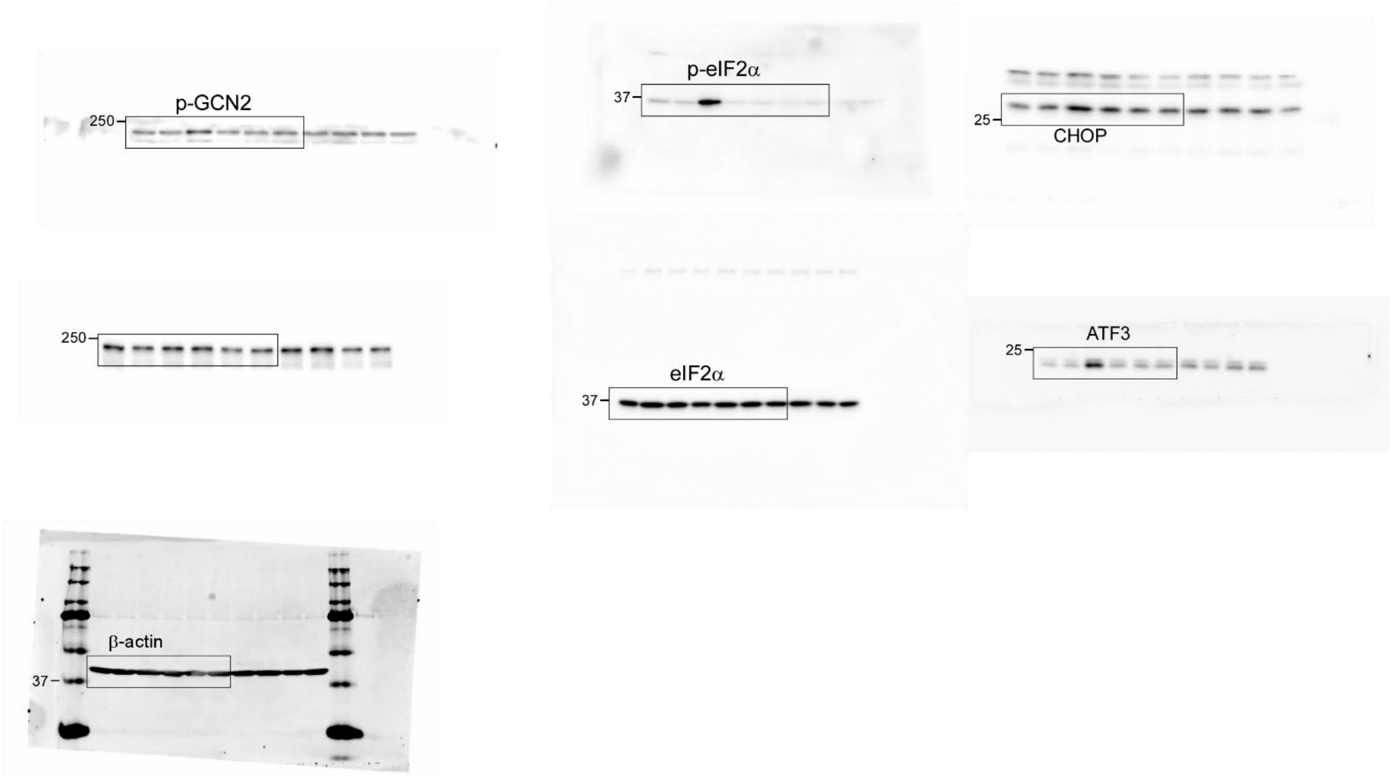

Uncropped blots in Supplementary figure 17 c.

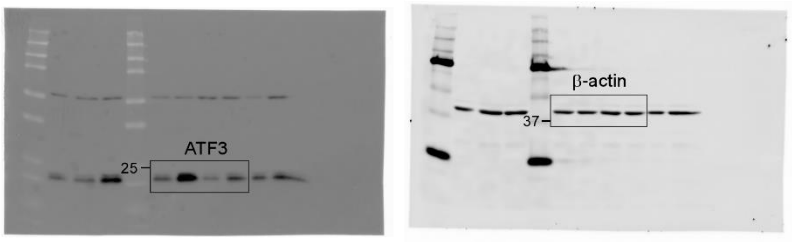

Uncropped blots in Supplementary figure 18.

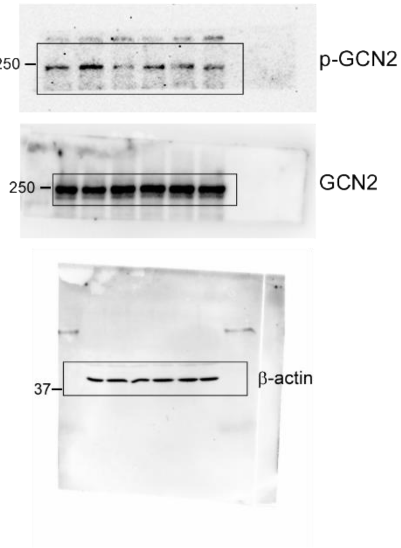

Supplement: Supplementary file 1 — Supplementary information [file 41467_2024_47190_MOESM1_ESM.pdf]
