# Supplementary material for: Mitochondrial injury induced by a Salmonella genotoxin triggers the proinflammatory senescence-associated secretory phenotype
Source: Nat Commun. 2024 Mar 30;15:2778. doi: 10.1038/s41467-024-47190-y (PMC10981749; doi:10.1038/s41467-024-47190-y)
Supplement: Supplementary file 3 — Description of Additional Supplementary Files [file 41467_2024_47190_MOESM3_ESM.pdf]

Description of Additional Supplementary Files

File Name: Supplementary Data 1

Description: All the plasmids used in the study.

File Name: Supplementary Data 2

Description: All antibodies and reagents used in the study are listed with their respective sources and identifiers.

File Name: Supplementary Data 3

Description: The oligonucleotide sequences of all primers and sgRNAs are provided in this study.
