# Supplementary material for: Mitochondrial injury induced by a Salmonella genotoxin triggers the proinflammatory senescence-associated secretory phenotype
Source: Nat Commun. 2024 Mar 30;15:2778. doi: 10.1038/s41467-024-47190-y (PMC10981749; doi:10.1038/s41467-024-47190-y)
Supplement: Supplementary file 7 — Reporting Summary [file 41467_2024_47190_MOESM7_ESM.pdf]

nature portfolio

Corresponding author(s): Shu-JungChang
Last updated by author(s): Mar.19th,2024

Reporting Summary

Nature Portfolio wishes to improve the reproducibility of the work that we publish. This form provides structure for consistency and transparency in reporting. For further information on Nature Portfolio policies, see our Editorial Policies and the Editorial Policy Checklist.

Please do not complete any field with "not applicable" or n/a. Refer to the help text for what text to use if an item is not relevant to your study.
For final submission: please carefully check your responses for accuracy; you will not be able to make changes later.

Statistics

For all statistical analyses, confirm that the following items are present in the figure legend, table legend, main text, or Methods section.

n/a	Confirmed	
☐	☒	The exact sample size (n) for each experimental group/condition, given as a discrete number and unit of measurement
☐	☒	A statement on whether measurements were taken from distinct samples or whether the same sample was measured repeatedly
☐	☒	The statistical test(s) used AND whether they are one- or two-sided<br>Only common tests should be described solely by name; describe more complex techniques in the Methods section.
☒	☐	A description of all covariates tested
☐	☒	A description of any assumptions or corrections, such as tests of normality and adjustment for multiple comparisons
☐	☒	A full description of the statistical parameters including central tendency (e.g. means) or other basic estimates (e.g. regression coefficient) AND variation (e.g. standard deviation) or associated estimates of uncertainty (e.g. confidence intervals)
☐	☒	For null hypothesis testing, the test statistic (e.g. F, t, r) with confidence intervals, effect sizes, degrees of freedom and P value noted<br>Give P values as exact values whenever suitable.
☒	☐	For Bayesian analysis, information on the choice of priors and Markov chain Monte Carlo settings
☒	☐	For hierarchical and complex designs, identification of the appropriate level for tests and full reporting of outcomes
☒	☐	Estimates of effect sizes (e.g. Cohen's d, Pearson's r), indicating how they were calculated

Our web collection on statistics for biologists contains articles on many of the points above.

Software and code

Policy information about availability of computer code

Data collection	CFX Manager™ Software (gene expression), ChemiDoc MP Imaging System and Image Lab (imaging and quantification of the band intensity of western blot), Canvas X Draw & Adobe Photoshop (image preparation), FlowJo (analysis of flow cytometry data), CLC Genomics and MSigDB (RNA-seq analysis), BioRender.
Data analysis	Statistic calculations were performed using GraphPad Prism V9. Images were processed and rendered by Image J (Fiji), Imaris (Oxford) or Zen-blue edition (Zeiss).

For manuscripts utilizing custom algorithms or software that are central to the research but not yet described in published literature, software must be made available to editors and reviewers. We strongly encourage code deposition in a community repository (e.g. GitHub). See the Nature Portfolio guidelines for submitting code & software for further information.

Data

Policy information about availability of data

All manuscripts must include a data availability statement. This statement should provide the following information, where applicable:

- Accession codes, unique identifiers, or web links for publicly available datasets
- A description of any restrictions on data availability
- For clinical datasets or third party data, please ensure that the statement adheres to our policy

The data discussed in this publication have been deposited in NCBI's Gene Expression Omnibus and are accessible through GEO Series accession number GSE247045. Source data are provided with this paper.

Field-specific reporting

Please select the one below that is the best fit for your research. If you are not sure, read the appropriate sections before making your selection.

☒ Lifesciences ☐ Behavioural & social sciences ☐ Ecological, evolutionary & environmental sciences

For a reference copy of the document with all sections, see nature.com/documents/nr-reporting-summary-flat.pdf

Life sciences study design

All studies must disclose on these points even when the disclosure is negative.

Samplesize	Sample size was determined based on references or experiences.
Dataexclusions	No data was excluded.
Replication	At least three independent experiments were performed with similar results. The number (n) of biological replicates were indicated in the figure legends.
Randomization	In all cases, samples(animals, cultured cell lines, etc) were randomly assigned to the different experimental groups.
Blinding	N/A

Reporting for specific materials, systems and methods

We require information from authors about some types of materials, experimental systems and methods used in many studies. Here, indicate whether each material, system or method listed is relevant to your study. If you are not sure if a list item applies to your research, read the appropriate section before selecting a response.

Materials & experimental systems

n/a	Involved in the study	
☐	☒	Antibodies
☐	☒	Eukaryotic cell lines
☒	☐	Palaeontology and archaeology
☐	☒	Animals and other organisms
☒	☐	Clinical data
☒	☐	Dual use research of concern
☒	☐	Plants

Methods

n/a	Involved in the study	
☒	☐	ChIP-seq
☐	☒	Flow cytometry
☒	☐	MRI-based neuroimaging

Antibodies

Antibodiesused

Rabbit anti-ATF3; Abcam; Cat #: ab207434
Rabbit anti-COXIV; Cell Signaling; Cat #: 4850
Rabbit anti-TFAM; Cell Signaling; Cat #: 8076
Rabbit anti-p16 INK4A; Cell Signaling; Cat #: 80772
Mouse anti-dsDNA; Abcam; Cat #: ab27156
Rabbit anti-phospho-eIF2α(Ser51); Cell Signaling; Cat #: 3398
Rabbit anti-eIF2 α; Cell Signaling; Cat #: 5324
Rabbit anti-p44/42 MAPK(Erk1/2); Cell Signaling; Cat #: 4695
Rabbit anti-phospho- p44/42 MAPK(Erk1/2) (Thr202/Tyr204); Cell Signaling; Cat #: 4370
Rabbit anti-GCN2; Cell Signaling; Cat #: 65981
Rabbit anti-GCN2 (phosphos T899); Abcam; Cat #: ab75836
Mouse anti-SAPK/JNK(Thr183/Tyr185); Cell signaling; Cat #: 9255
Rabbit anti-SAPK/JNK; Cell Signaling; Cat #: 9252
Mouse anti-p38α; Cell Signaling; Cat #: 9217
Rabbit anti-phosphop38 MAPK (Thr180/Tyr182); Cell Signaling; Cat #: 4511
Rabbit anti-NF-κB p65; Cell Signaling; Cat #: 8242
Rabbit anti-phospho-NF-κB p65 (Ser536); Cell Signaling; Cat #: 3033
Mouse anti-TOMM20; Santa Cruz; Cat #: sc-17764
Rabbit anti-PERK; Cell signaling; Cat #: 5683
Rabbit anti-PKR (phosphor T446); Cell signaling; Cat #: ab32036
Mouse anti-PKR; Santa Cruz; Cat #: sc-6282
Rabbit anti-phospho TBK-1/NAK (Ser172); Cell signaling; Cat #: 5483
Rabbit anti-TBK-1/NAK; Cell signaling; Cat #: 3504
Anti-mouse IgG, HRP-linked antibody; Cell signaling; Cat #: 7076
Anti-rabbit IgG, HRP-linked antibody; Cell signaling; Cat #: 7074
StarBright Blue 700 goat anti-rabbit IgG; Bio-Rad; Cat #: 12004161
Peroxidase AffiniPure goat anti-mouse IgG (H+L); The Jackson Laboratory; Cat #: 115035003
Peroxidase AffiniPure goat anti-rabbit IgG (H+L); The Jackson Laboratory; Cat #: 111035003
Mouse anti-γH2AX; Merck Millipore; Cat #: 05636
Rabbit anti-γH2AX; Abcam; Cat #: ab11175
Rabbit anti-β-actin; ABclonal; Cat #: AC026
DAPI; Sigma-Aldrich; Cat #: D9542
Rabbit anti-GADD153; GeneTex; Cat #: GTX112827
Rabbit anti-STING; Cell signaling; Cat #: 13647
Rabbit anti-cGAS; Cell signaling; Cat #: 15102
Rabbit anti-p53; Cell signaling; Cat #: 9282
Normal mouse IgG; Santa Cruz; Cat #: sc-2025
Goat anti-rabbit IgG (H+L) cross absorbed secondary antibody, Biotin-XX; Thermo Fisher; Cat #: B2770
6x-His Tag Monoclonal Antibody; Thermo Fisher; Cat #: 21315

Mouse Phospho-p53; Cell signaling; Cat #: 9286
Rabbit anti-p21; Cell signaling; Cat #: 2947
Rabbit anti-AIM2; GeneTex; Cat #: GTX116487
Rabbit anti-ATM; GeneTex; Cat #: GTX111106
Mouse anti-CD16/32; BioLegend; Cat #: 101319
Mouse anti-Caspase 9; Cell signaling; Cat #: 9508
Mouse anti-NLRP3; AdipoGen; Cat #: AG-20B-0014
Mouse anti-CD289 (TLR9) FITC; eBioscience; Cat #: 11-9093-80
InVivoMAb anti-mouse CD3ε (145-2C11); Bio X Cell ; Cat #: BE0001
InVivoMAb anti-mouse CD28 (37.51); Bio X Cell; Cat #: BE0015-1
Goat Anti-Mouse Ig (H+L); Southern Biotech; Cat #: 1010-01
APC/FireTM 750 anti-mouse CD4; Biolegend; Cat #: 100568 Rabbit
anti-GSDMD; Cell signaling; Cat #: 96458
PE Anti-mouse Granzyme B (NGZB); eBioscience; Cat #: 12-8898-80
Alexa Fluor 700 anti- mouse CD8a; BD Pharmingen; Cat #: 557959
PE/Dazzle anti-mouse IFN-g (XMG1.2); Biolegend; Cat#: 505845
FITC anti-mouse CD28 (E18); Biolegend; Cat #: 122007
APC anti-mouse TIGIT (1G9); Biolegend; Cat #: 142105
Brilliant Violet 605 anti-mouse/human KLRG-1 (2F1/KLRG1); Biolegend; Cat #: 138419
Rabbit anti-phospho STING (Ser366); Cell signaling; Cat #: 50907
Streptavidin, Alexa Flour 568 conjugate; Thermo Fisher; Cat #: S11226
Goat anti-Mouse IgG1 Cross-Adsorbed Secondary Antibody, Alexa Fluor™ 594; Invitrogen; Cat#: A-21125
Goat anti-Rabbit IgG (H+L) Highly Cross-Adsorbed Secondary Antibody, Alexa Fluor™ 488; Invitrogen; Cat#: A-11034
Goat anti-Rabbit IgG (H+L) Highly Cross-Adsorbed Secondary Antibody, Alexa Fluor™ 647; invitrogen; Cat#: A-11012
Goat anti-Rabbit IgG (H+L) Cross-Adsorbed Secondary Antibody, Alexa Fluor™ 594; invitrogen; Cat#: A-11012
Goat anti-Mouse IgG (H+L) Highly Cross-Adsorbed Secondary Antibody, Alexa Fluor™ Plus 647; Invitrogen; Cat#: A-32728
Goat anti-Mouse IgG1 Cross-Adsorbed Secondary Antibody, Alexa Fluor™ 488; Invitrogen; Cat#: A-21121

Validation

All antibodies were purchased from reputable vendors and validated with positive or negative control in this study. Commercial antibodieswere also validated by the manufacturers as stated in the manual instructions.

Eukaryotic cell lines

Policy information about cell lines and Sex and Gender in Research

Cell line source(s)

Henle-407 originally obtained from the Roy Curtiss laboratory collection, were provided as a gift from Dr. Jorge Galan's lab.
THP-1 ATCC TIB-202, RAW 264.7 ATCC TIB-71

Authentication

Cell lines were not authenticated

Mycoplasmacontamination

The cell lines were routinely tested with Mycoplasma.

Commonlymisidentifiedlines
(See ICLAC register)

N/A

Animals and other organisms

Policy information about studies involving animals; ARRIVE guidelines recommended for reporting animal research

Laboratoryanimals

Six- to eight-week-old, sex-matched and wild-type C57BL/6 mice were used in related experiments.

Wildanimals

N/A

Field-collectedsamples

N/A

Ethicsoversight

All animal experiments were conducted in strict accordance with the protocols approved by the policies of the National Taiwan University College of Medicine

Note that full information on the approval of the study protocol must also be provided in the manuscript.

Flow Cytometry

Plots

Confirmthat:

☒ The axis labels state the marker and fluorochrome used (e.g. CD4-FITC).

☒ The axis scales are clearly visible. Include numbers along axes only for bottom left plot of group (a 'group' is an analysis of identical markers).

☒ All plots are contour plots with outliers or pseudocolor plots.

☒ A numerical value for number of cells or percentage (with statistics) is provided.

Methodology

Samplepreparation

All protocols of the FACS related experiments are described in detail in the "Methods" section. Typically, the FACS experiments involved staining with one to two colors, unless explicitly specified otherwise.

Instrument

Flow data was obtained by FACSCalibur Flow Cytometer (BD Biosciences)

Software

Data were analyzed with Flowjo V10.8.1.

Cell populationabundance

Primary murine CD8/CD4 T cells, THP-1 and Henle-407 cells

THP-1 and Henle-407 populations were identified with FSC-H/SSC-H. The gating strategies of CD4/CD8 T cell related experiments were

Gatingstrategy

☒ Tick this box to confirm that a figure exemplifying the gating strategy is provided in the Supplementary Information
